# Eucalyptol ameliorates early brain injury after subarachnoid haemorrhage via antioxidant and anti-inflammatory effects in a rat model

**DOI:** 10.1080/13880209.2021.1876101

**Published:** 2021-02-08

**Authors:** Gang Xu, Junsheng Guo, Chunming Sun

**Affiliations:** Department of Neurosurgery, Liyang People’s Hospital, Affiliated Hospital of Nantong University, Changzhou, China

**Keywords:** Inflammation, neurological function, Nissl body

## Abstract

**Context:**

As the terpenoid oxide extracted from *Eucalyptus* L. Herit (Myrtaceae), eucalyptol (EUC) has anti-inflammatory and antioxidant effects.

**Objective:**

To evaluate the neuroprotective role of EUC in subarachnoid haemorrhage (SAH).

**Materials and methods:**

Sprague-Dawley rats were divided into 4 groups: sham group, SAH group, SAH + vehicle group, and SAH + EUC group. SAH was induced by endovascular perforation. In SAH + EUC group, 100 mg/kg EUC was administrated intraperitoneally at 1 h before SAH and 30 min after SAH, respectively. Neurological deficits were examined by modified Neurological Severity Scores (mNSS). The brain edoema was evaluated by wet-dry method. Neuronal apoptosis was detected by Nissl staining. The expression of Bcl-2, cleaved caspase-3, phospho-NF-κB p65, ionised calcium-binding adapter molecule-1 (Iba-1), nuclear factor erythroid-2 (Nrf-2), and haem oxygenase 1 (HO-1) were measured by Western blot. Expression of pro-inflammatory cytokines was detected by qRT-PCR. Oxidative stress markers were also measured.

**Results:**

EUC markedly relieved brain edoema (from 81.22% to 78.33%) and neurological deficits [from 16.28 to 9.28 (24 h); from 12.50 to 7.58 (48 h)]. EUC reduced neuronal apoptosis, microglial activation, and oxidative stress. EUC increased the expression of HO-1 (1.15-fold), Nrf2 (1.34-fold) and Bcl-2 (1.17-fold) in the rats’ brain tissue, and down-regulated the expressions of cleaved caspase-3 (41.09%), phospho-NF-κB p65 (14.38%) and pro-inflammatory cytokines [TNF-α (34.33%), IL-1β (50.40%) and IL-6 (59.13%)].

**Discussion and Conclusion:**

For the first time, this study confirms that EUC has neuroprotective effects against early brain injury after experimental SAH in rats.

## Introduction

With a high morbidity and mortality, subarachnoid haemorrhage (SAH) is mainly caused by an intracranial ruptured aneurysm, which is related to intracranial hypertension (Macdonald 2014). Early brain injury (EBI) refers to the pathological processes that emerge within 72 h after SAH, characterised by elevated intracranial pressure, reduced cerebral blood flow and cerebral perfusion pressure, dysregulation of cellular Ca^2+^ homeostasis, damage of blood-brain barrier (BBB), acute cerebral vasospasm, brain edoema, neuroinflammation, and apoptosis of neurons. EBI is the determinant of neurological dysfunction and poor prognosis of the patients (Hankey 2006; Chen et al. 2014).

The apoptosis of neurons is found in SAH patients and animal models (Wang et al. 2015). There is also evidence showing that apoptotic neurons after SAH contribute to neurological deficits, and anti-apoptotic drugs can probably attenuate EBI after SAH. Also, neuroinflammation is a pivotal pathological factor in EBI (He et al. 2015; Zhang et al. 2015). Nuclear factor kappa B (NF-κB) signalling is involved in microglia polarisation, and promoting the secretion of pro-inflammatory cytokines such as TNF-α, IL-1β, and IL-6 (Lawrence 2009). Ionised calcium-binding adapter molecule-1 (Iba-1) is a microglia activation marker. In addition, it is reported that excessive reactive nitrogen species and reactive oxygen species (ROS) are produced in the early stage after SAH, such as hydroxyl radicals, nitric oxide, peroxynitrite, superoxide anions, and hydrogen peroxide, which damage the defense system of antioxidants, and they are closely associated with EBI and secondary neurological injury (Ostrowski et al. 2006; Sehba et al. 2012). Reducing neuronal apoptosis, inhibiting inflammation and oxidative stress during EBI can potentially improve the neurological recovery after SAH.

As a kind of terpenoid oxide, eucalyptol (1,8-cineole, EUC) is extracted from *Eucalyptus* L. Herit (Myrtaceae), such as *Eucalyptus globules* Labill and *Eucalyptus tereticornis* Sm. Due to its anti-inflammatory, antioxidative and antibacterial characteristics, it is used as a pain relief drug and as a treatment for inflammatory airway diseases (Bastos et al. 2009; Rahimi-Nasrabadi et al. 2012; Juergens 2014; Caceres et al. 2017). Recently, EUC treatment has been proved to reduce LPS-induced lung inflammatory injury in mice (Zhao et al. 2014). Interestingly, EUC, which can easily pass through BBB, may have a direct regulatory function on brain’s receptors and enzymes (Moss and Oliver 2012). Nevertheless, it remains unknown whether EUC has neuroprotective function in EBI after SAH.

In this study, we established a rat model with EBI after SAH to explore whether EUC had the function to ameliorate the EBI and improve the neurological function after SAH. It was proved that EUC was a promising drug to reduce neuronal apoptosis, neuroinflammation and attenuate oxidative stress responses in the brain, which suggested that EUC had the potential to treat EBI after SAH.

## Materials and methods

### Animals

The experimental procedures were approved by the Experimental Animal Welfare Committee of the Affiliated Hospital of Nantong University (Approval No. 2018011), following the Animal Research: Reporting of *In Vivo* Experiments (AARIVE) guidelines, issued by National Centre for the Replacement Refinement & Reduction of Animals in Research. Eighty male Sprague Dawley (SD) rats (250–300 g) were available from the Experimental Animal Centre, Affiliated Hospital of Nantong University. The animals were placed under 12 h light/dark cycle with standard temperature (23 °C) and humidity and sufficient water and food. The rats were randomly divided into 4 groups (*n* = 20 in each group): (1) sham group; (2) SAH group; (3) SAH + vehicle group; (4) SAH + EUC group. SAH rat model in which rats underwent intravascular perforation was established according to the protocol provided by the previous study (Li et al., 2016).

**Figure 1.**
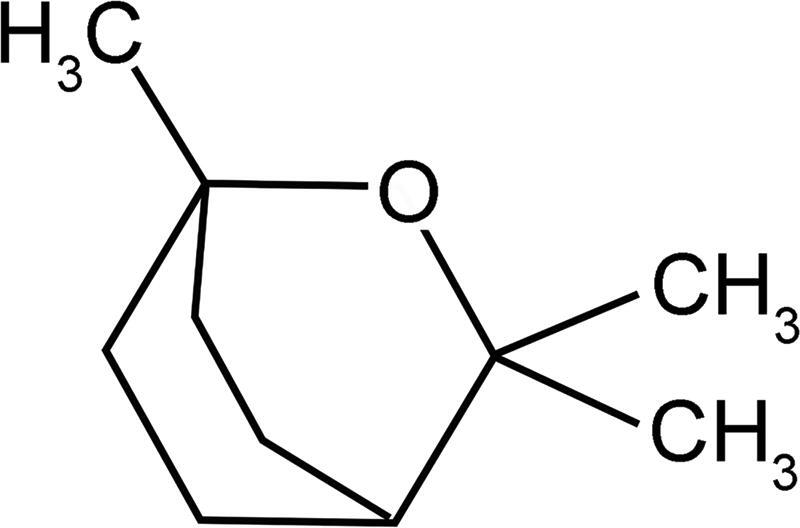
EUC’s molecular structure.

In brief, rats anaesthetised with pentobarbital sodium (50 mg/kg) were put on a heating blanket to keep their rectal temperature at 37 ± 0.5 °C. The left carotid artery and its branches were isolated. A blunted 4–0 monofilament nylon suture was inserted into the right external carotid artery and then it was pushed into the internal carotid artery until there was resistance (about 18 mm). Next, it was further advanced 3 mm to penetrate the artery. Next, the suture was withdrawn to allow blood reperfusion to induce SAH. In the sham group, the rats were treated in the same way but the arteries were not punctured. After the operation, the rats were put back into cages for recovery. The molecular structure of EUC was shown in Figure 1. EUC (purity, ≥99%; Sigma, St. Louis, MO, USA) was dissolved into corn oil, and then the intraperitoneal injection (100 mg/kg) was performed 1 h before SAH and 30 min after SAH, respectively.

### Evaluation of neurological deficit

Modified Neurological Severity Scores (mNSS) tests were performed 24 and 48 h after SAH by an investigator blind to the experimental conditions and grouping according to the protocol previously reported (Chen et al. 2001). Briefly, the animals’ motor, sense, balance and reflex were scored with a range of 0–18 points. 0, no neurological deficit which meant that the rat was totally normal; 1–6, mild injury; 7–12, moderate injury; 13–17, severe injury; 18 loss of consciousness and death.

### Evaluation of brain edoema

Rat brain water content was determined by the wet-dry weighting method. The rat brain hemisphere with injury was taken out and weighed quickly, then it was placed in an oven at 105 °C and dried for 24 h until the weight remained unchanged, and then the dry weight of the brain tissue was weighed. The formula for how to obtain the value of brain water content was: brain water content (%) = (wet weight - dry weight)/wet weight × 100%.

### Nissl staining

The neuronal survival rate was assayed employing Nissl staining at 24 h after SAH. Rat brains were fixed for 1 h by transcranial perfusion with 4% paraformaldehyde. Paraffin-embedded hippocampal tissue slices (with a thickness of 4 μm) were stained for about 30 min in 1% toluidine blue solution (Beyotime, Shanghai, China) and washed with double distilled water, and the colours were separated by 2% hydrochloric acidic alcohol. Then the slices were dehydrated with 95% ethanol for 2 min, incubated with dimethylbenzene for 3 min, and sealed with neutral balsam. Slices were then observed under a microscope, and the photographs were taken. With relatively large cell bodies and round nuclei, normal neurons were rich in cytoplasm, while the damaged cells had a contracted cell body, pyknotic nuclei, dark cytoplasm, and many vacuoles. The diagnosis and cell count were carried out by an investigator blind to the experiments.

### Measurement of malonyldialdehyde (MDA), superoxide dismutase (SOD) and glutathione peroxidase (GSH-Px)

Oxidative stress markers, including MDA, SOD and GSH-Px, were detected and analysed with commercial kits (Solarbio, Beijing, China). In short, the hippocampal tissue of the rats in each group was collected on ice, and the hippocampal homogenate was prepared with normal saline, and centrifuged at 12,000 *g* for 10 min at 4 °C, and the supernatant was taken. The absorbance (A value) of the sample was measured at 450, 532, and 600 nm using a microplate reader. MDA content (nmol/mg) = 6.45 × (A_532nm_ - A_600nm_) − 0.56 × A_4500nm_. SOD activity (U/mg) (absorbance) was measured at 560 nm using a microplate reader. GSH-Px activity (U/mg) (absorbance) was determined at 412 nm using a microplate reader. The experimental procedures strictly followed the manufacturer’s instructions.

### Quantitative real-time polymerase chain reaction (qRT-PCR)

The total RNA was extracted from rat brain tissues using TRIzol reagent (Invitrogen, Carlsbad, CA, USA), and then the PrimeScript™ RT reagent Kit with gDNA Eraser (Takara, Dalian, China) was employed to reversely transcribe the total RNA into cDNA. The SYBR^®^ Premix Ex Taq™ II (Takara, Dalian, China) was applied for DNA amplification to detect the transcription levels of inflammatory factors IL-1β, TNF-α and IL-6. The target genes and glyceraldehyde-3-phosphate dehydrogenase (GAPDH; internal reference gene) were amplified in each sample, and 2^−ΔΔCT^ method was utilised to analyse the relative expression of the target genes. The primer sequences were as follows: TNF-α: forward: 5′-CCCTCACACTCAGATCATCTTCT-3′ and reverse: 5′-GCTACGACGTGGGCTACAG-3′; IL-6: forward: 5′-TAGTCCTTCCTACCCCAATTTCC-3′ and reverse: 5′-TGGTCCTTAGCCACTCCTTC-3′; IL-1β: forward: 5′-GCAACTGTTCCTGAACTCAACT-3′ and reverse: 5′-ATCTTTTGGGGTCCGTCAACT-3′; GAPDH: forward: 5′-TCATCCCAGAGCTGAACG-3′ and reverse: 5′-TCATACTTGGCAGGTTTCTCC-3′.

### Western blot assay

In each group, the total protein in the brain tissue of the rats was extracted using RIPA buffer (Beyotime, Jiangsu, China) and the bicinchoninic acid (BCA) method was adopted to determine the protein concentration of each group. After denaturation, 20 μg of protein was put into each well, and then the samples were separated on 10% sodium dodecyl sulfate-polyacrylamide gel electrophoresis (SDS-PAGE). After that, the protein was transferred onto a polyvinylidene fluoride (PVDF) membrane (Millipore, Bedford, MA, USA), followed by being blocked for 1 h with 5% skim milk at room temperature, and then the membrane was incubated at 4 °C overnight with primary antibodies against Bcl-2 (1:500; ab185002; Abcam), cleaved caspase-3 (1:500; ab2302; Abcam), Iba-1 (1:500; ab5076; Abcam), NF-κB p-p65 (S536) (1:500; ab86299; Abcam), nuclear factor erythroid-2 (Nrf-2) (1:500; ab62352; Abcam), haem oxygenase 1 (HO-1) (1:500; ab204524; Abcam) and GAPDH (1:2000; ab181602; Abcam). After that, the membrane was rinsed with TBST 2 times (5 min each time), incubated with corresponding secondary antibody, goat anti-mouse/rabbit IgG antibody HRP (Bioworld, Minneapolis, MN, USA), for 1.5 h at room temperature, and then washed 3 times (5 min each time) with TBST. Ultimately, the ECL chemiluminescence kit (Beyotime, Shanghai, China) was used for the colour development. The image analysis software ImageJ (NIH Image, Bethesda, MD, USA) was employed to perform the analysis of grey values of the bands.

### Statistical analysis

Data analysis was carried out with SPSS 24.0 (SPSS Inc., Chicago, IL, USA). Mean ± SD was used to express all the data. The difference between the two groups was tested with Student’s *t*-test. GraphPad Prism 8.0 (GraphPad Software, San Diego, CA, USA) was employed to draw the graphics, and values with *p* < 0.05 were considered to be of statistical significance.

## Results

### Mortality in rats after SAH

During the experiment, all rats’ body temperature, body weight, and blood pressure were stable (data are not shown). The mortality of the rats was 0% (0/20), 20.0% (4/20), 15.0% (0/20) and 15.0% (3/20) in the sham group, SAH group, SAH + vehicle group and SAH + EUC group, respectively.

### EUC ameliorated SAH-induced neurological deficit and brain edoema

**Figure 2.**
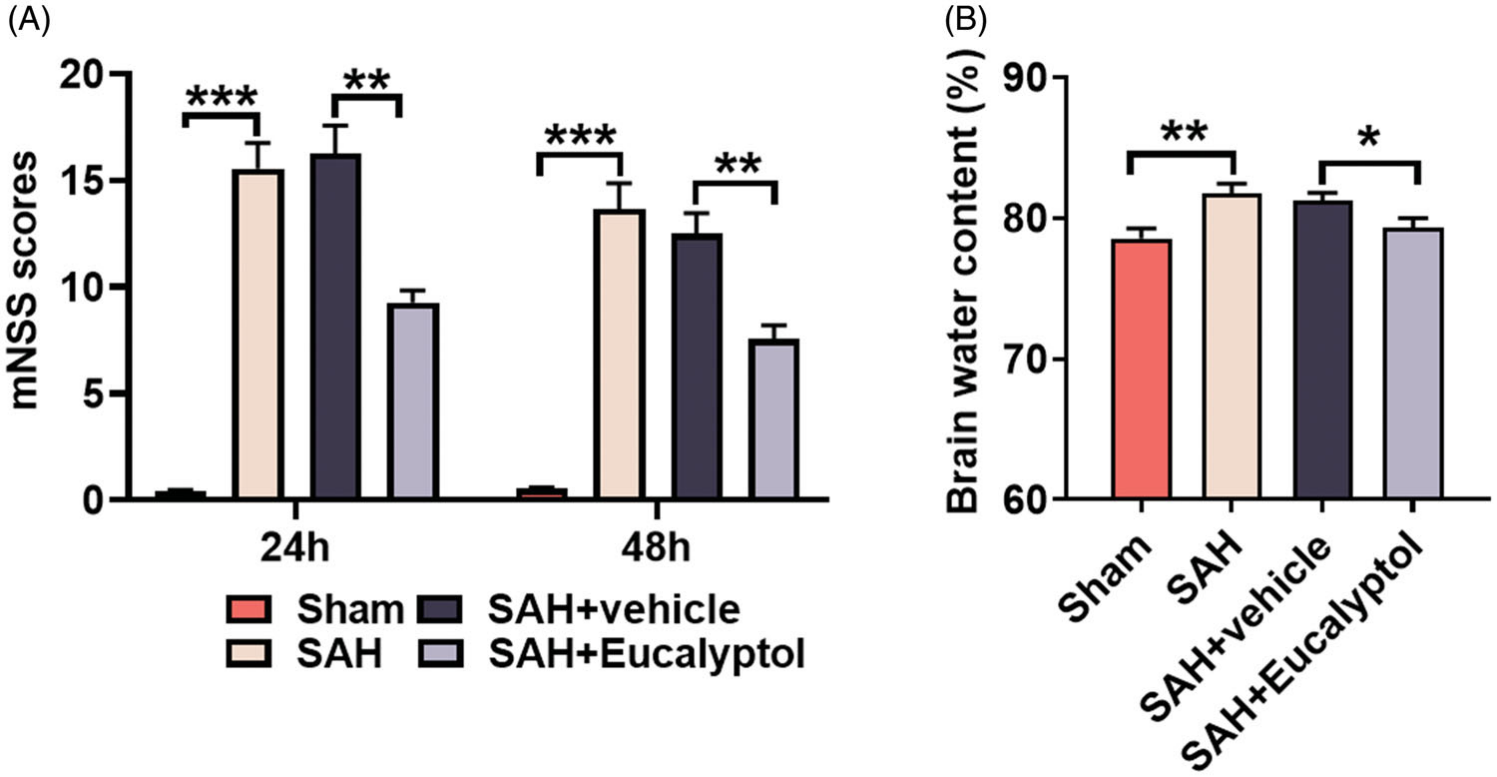
EUC ameliorated SAH-induced neurological deficits and brain edoema. EUC was dissolved into corn oil, and then the intraperitoneal injection (100 mg/kg) was performed 1 h before SAH and 30 min after SAH, respectively. (A) mNSS was adopted to evaluate neurological deficits of the rats at 24 and 48 h after SAH (*n* = 5); (B) Wet-dry method was employed to detect brain edoema in rats of each group (*n* = 3). *p < 0.05, **p < 0.01 and ***p < 0.001.

To determine EUC’s effect on brain injury of rats after SAH, we examined the neurological deficit of rats using mNSS method and brain edoema was evaluated employing wet-dry method. mNSS showed that neurological function of rats in SAH group and SAH + vehicle group was severely impaired compared with that in the sham group, but neurological function of the rats treated with EUC was significantly improved [from 16.28 to 9.28 (24 h), *p* < 0.05; from 12.50 to 7.58 (48 h), *p* < 0.05] (Figure 2(A)). Additionally, the brain water content of the rats in SAH group, in comparison with that in the sham group, was increased significantly; EUC treatment, in comparison with in the SAH + vehicle group, markedly reduced the brain water content of the rats (from 81.22% to 78.33%, *p* < 0.05) (Figure 2(B)). The results indicated that EUC treatment could improve the neurological function and reduce brain edoema of the rats after SAH.

### Effects of EUC on SAH-induced neuronal apoptosis

**Figure 3.**
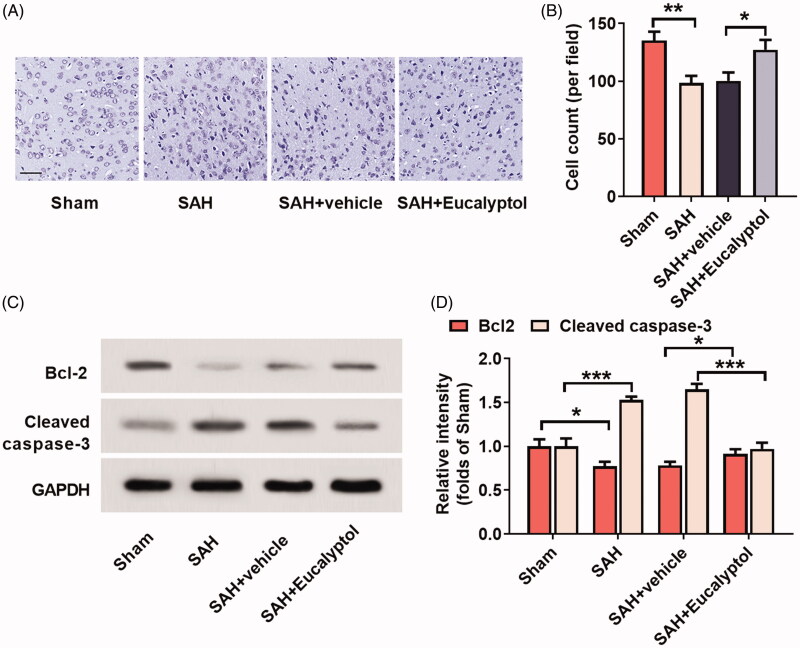
EUC’s effects on SAH-induced neuronal apoptosis. EUC was dissolved into corn oil, and then the intraperitoneal injection (100 mg/kg) was performed 1 h before SAH and 30 min after SAH, respectively. (A,B) Nissl staining was utilised to test neuronal apoptosis in the rats of each group (*n* = 3), Bar = 50 μm; (C,D) Western blot analysis was utilised to test the protein expressions of cleaved caspase-3 and Bcl-2 (*n* = 3). *p < 0.05, **p < 0.01 and ***p < 0.001.

In this work, Nissl staining was employed to stain rat brain tissue in each group to detect the neuronal apoptosis 24 h after SAH. The results revealed that the cells in the sham group were regularly arranged with clear nuclei and uniform staining, while in the SAH group, the cells were severely damaged and disorderly arranged with pyknotic and hyperchromatic nuclei; in SAH + EUC group, the cell morphology was relatively intact, the number of Nissl bodies was increased, and the staining was well-distributed, compared with those in SAH + vehicle group (Figure 3(A,B)). This suggested that EUC administration significantly reduced the damage caused by SAH to neurons of rats. Western blot was utilised to further analyse the expression of apoptosis-related proteins cleaved caspase-3 and Bcl-2 in brain tissues of the rats in each group. The results unveiled that EUC treatment significantly up-regulated the expression of Bcl-2 (1.17-fold, *p* < 0.05) and down-regulated the expression of cleaved caspase-3 (41.09%, *p* < 0.05) (vs. SAH + vehicle group) (Figure 3(C,D)). Collectively, these results indicated that EUC could reduce the apoptosis of neurons to inhibit EBI after SAH.

### EUC’s effects on SAH-induced activation of microglia

**Figure 4.**
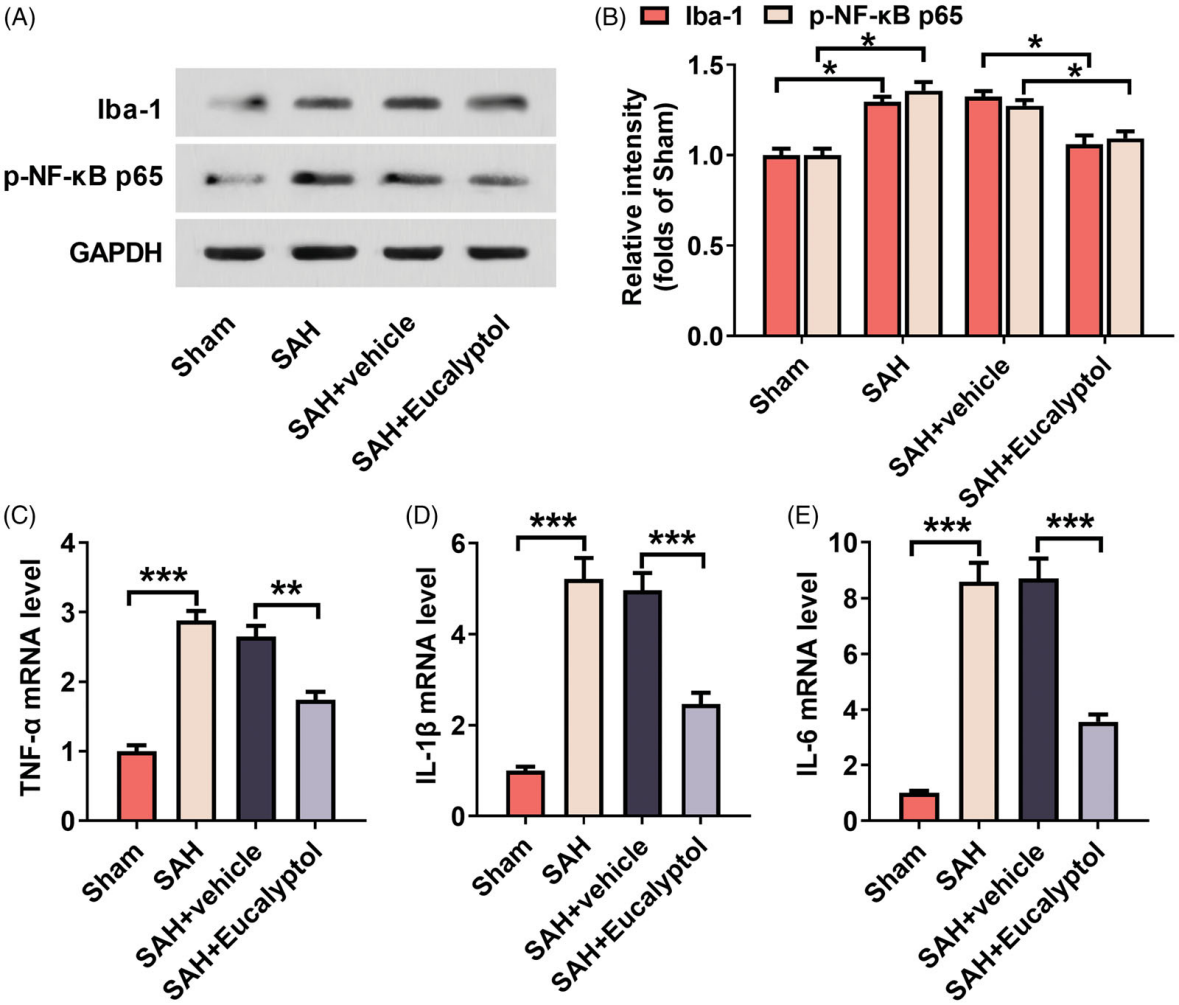
EUC’s effects on SAH-induced activation of microglial cells. EUC was dissolved into corn oil, and then the intraperitoneal injection (100 mg/kg) was performed 1 h before SAH and 30 min after SAH, respectively. (A,B) Western blot analysis was utilised to test the protein expression of Iba-1 and p-NF-κB p-65 in brain tissues of rats in each group (*n* = 3); (C–E) qRT-PCR was utilised to detect the relative expression of mRNAs of inflammatory cytokines TNF-α, IL-6 and IL-1β in brain tissues of the rats (*n* = 3). *p < 0.05, **p < 0.01 and ***p < 0.001.

The expression of phospho-NF-κB p65 and Iba-1 in brain tissues of the rats was analysed by Western blot, the results of which demonstrated that the expression levels of phospho-NF-κB p65 and Iba-1 were relatively low in the sham group; compared to in the sham group, SAH significantly elevated the expressions of phospho-NF-κB p65 and Iba-1, suggesting microglia activation and neuroinflammation in the brains. However, the expression of phospho-NF-κB p65 (14.38%, *p* < 0.05) and Iba-1 (18.42%, *p* < 0.05) was markedly down-regulated after EUC treatment (Figure 4(A,B)). EUC’s effect on the expression of inflammatory cytokines was subsequently assayed by qRT-PCR. As shown, compared to in the rats in SAH + vehicle group, the administration of EUC markedly reduced the expression of TNF-α (34.33%, *p* < 0.05), IL-1β (50.40%, *p* < 0.05) and IL-6 (59.13%, *p* < 0.05) mRNAs in the brain tissues of the rats (Figure 4(C–E)).

### EUC’s effects on SAH-induced oxidative stress

**Figure 5.**
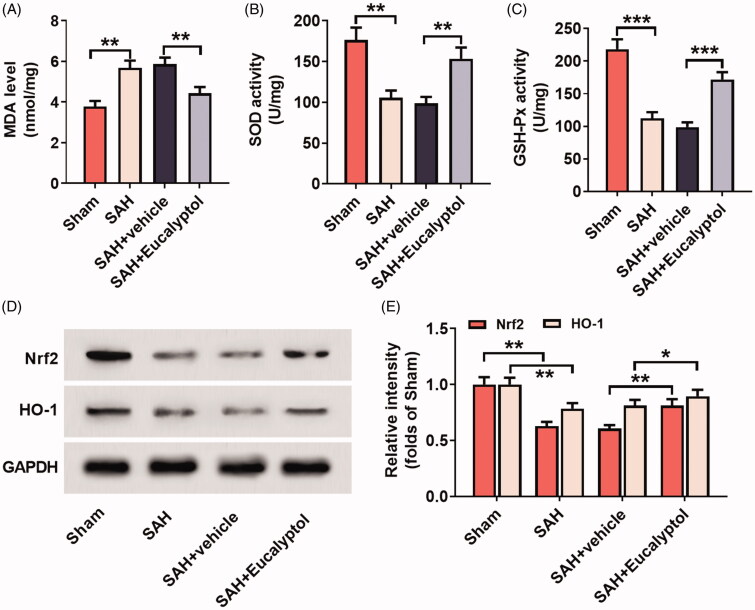
EUC’s effects on SAH-induced oxidative stress. EUC was dissolved into corn oil, and then the intraperitoneal injection (100 mg/kg) was performed 1 h before SAH and 30 min after SAH, respectively. (A–C) The MDA content, SOD and GSH-Px activity in brain tissues in rats of each group was determined (*n* = 3); (D–E) Western blot analysis was employed to detect the protein expression of HO-1 and Nrf2 in brain tissue of the rats of each group (*n* = 3). *p < 0.05, **p < 0.01 and ***p < 0.001.

To pinpoint EUC’s effect on oxidative stress induced by SAH, in this research, the markers of oxidative stress responses in the brain tissues of the rats in each group were detected. Compared with the sham group, MDA content was markedly increased and SOD and GSH-Px activity was significantly reduced in the brain tissue of rats in SAH group; while SOD and GSH-Px activities after EUC treatment were significantly elevated and MDA content was reduced (vs. SAH + vehicle group) (Figure 5(A–C)). These results suggested that EUC could markedly attenuate the oxidative stress response induced by SAH. Western blot was further used to analyse the change of the activation of Nrf2/HO-1 antioxidant pathway. As shown, EUC treatment markedly up-regulated the expressions of Nrf2 (1.34-fold, *p* < 0.05) and HO-1 (1.15-fold, *p* < 0.05) in the brain tissue of rats (Figure 5(D,E)). This suggested that EUC could activate the Nrf2/HO-1 signalling cascade and reduce SAH-induced oxidative stress response.

## Discussion

Each year, about 10 cases per 100,000 people suffer from SAH and most of the patients are middle-aged, which places a huge burden on the economy and society (Solár et al. 2020). EBI accounts for 60% of SAH-related deaths, so ameliorating EBI is the main goal in SAH patient care in clinical practice (Li et al. 2019). Therapies that alleviate EBI can probably reduce the mortality and disability of patients with SAH. In the present study, we demonstrated that EUC was a promising drug to attenuate EBI after SAH in a rat model.

Bcl-2/Bax/caspase-3 apoptosis signalling is a crucial regulator in cell survival and apoptosis, and targeting this pathway is considered as a potential strategy to reduce the apoptosis of neurons in a variety of neurological diseases. For instance, in a rat model, tetramethylpyrazine inhibits cerebral vasospasm and EBI of the rats with SAH via inhibiting caspase-3 dependent proapoptosis pathway (Gao et al. 2008); paeoniflorin can probably exert neuroprotective effects in Parkinson's disease partly via up-regulating Bcl-2 and inhibiting caspase-3 (Zheng et al. 2017); in a rat model with SAH, apelin-13 inhibits EBI through regulating GLP-1R/PI3K/Akt signalling, accompanied with increased expression of Bcl-2 and reduced expression of Bax and caspase-3 (Liu et al. 2019). In this research, we demonstrated that EUC treatment markedly reduced neuronal apoptosis of rats after SAH; mechanistically, EUC down-regulated the expression of cleaved caspase-3 and up-regulated the expression of Bcl-2. To our best knowledge, this work is the first to validate the neuroprotective function of ECU in SAH, which is consistent with its beneficial role for neurons in animal model with ischaemic stroke (Ryu et al. 2014).

In the early stage after SAH, activated microglial cells and astrocytes are beneficial. Nevertheless, excessive activation of microglia leads to the secretion of inflammatory factors to aggravate brain injury. Therefore, the inhibition of the excessive activation of microglial cells can alleviate EBI after SAH (Murakami et al. 2011; Hanafy 2013; Helbok et al. 2015). We found in this research that microglia activation and the expression of pro-inflammatory cytokines were increased significantly in the early stage of SAH, resulting in severe brain injury, which is consistent with previous reports. Importantly, it was demonstrated that EUC could significantly impede the activation of SAH-induced activation of microglia and reduce the expressions of phospho-NF-κB p65 and Iba-1, accompanied with reduced expression levels of inflammatory factors IL-6, IL-1β and TNF-α, which suggested that the microglial activation was suppressed and neuroinflammation was inhibited. It was concluded that EUC could reduce the inflammatory response via inhibiting microglial activation and NF-κB signalling to ameliorate EBI after SAH.

Growing evidence shows that in the pathogenesis of EBI, oxidative stress figures prominently (Ayer and Zhang 2008). Previous studies report that under oxidative stress, Nrf2 can be transferred from cytoplasm to nucleus and activate the transcription of multiple antioxidant genes (Itoh et al. 2004; Zhang et al. 2010). Nrf2 is proved to reduce the brain’s oxidative stress via inducing detoxifying enzymes and antioxidants in traumatic brain injury, cerebral ischaemia and EBI after SAH (Gilgun-Sherki et al. 2002; van Muiswinkel and Kuiperij 2005; Chen et al. 2011). Activating Nrf2 is considered as a promising strategy to ameliorate neurological deficit and to improve functional recovery after brain injury. For example, aloperine treatment in the SAH rat model can inhibit oxidative stress, reduce the permeability of BBB and reduce neuron apoptosis through the Nrf2 pathway (Song et al. 2018). We found in the study that EUC significantly suppressed the accumulation of MDA and increased the activity of SOD and GSH-Px, suggesting that EUC could attenuate SAH-induced oxidative stress response. It was also demonstrated that EUC treatment could increase the expression of Nrf2 and HO-1 in brain tissues of the rats, providing evidence that EUC might relieve SAH-induced oxidative stress response and ameliorate EBI via activating Nrf2/HO-1 pathway.

This study proves that EUC can attenuate EBI after SAH in a rat model, providing the theoretical basis for its clinical application as a neuroprotective drug. Notably, in the future, further research is still necessary to reveal the exact mechanism of its characteristics of anti-apoptosis, anti-inflammation and antioxidation. What’s more, this work is only a preclinical study, and more pharmacological tests should be performed before its application in clinic.

## Conclusion

Our research shows that EUC plays a neuroprotective role to ameliorate EBI after SAH. It is revealed that EUC can reduce neuronal apoptosis, inhibit neuroinflammation, block oxidative stress responses through multiple mechanisms, and has the potential to be a novel drug for clinical treatment of EBI after SAH.

## Data Availability

The data used to support the findings of this study are available from the corresponding author upon request.
